# Supplementary material for: Tuning the Fröhlich exciton-phonon scattering in monolayer MoS2
Source: Nat Commun. 2019 Feb 18;10:807. doi: 10.1038/s41467-019-08764-3 (PMC6379367; doi:10.1038/s41467-019-08764-3)
Supplement: Supplementary file 1 — Supplementary Information [file 41467_2019_8764_MOESM1_ESM.pdf]

Supplementary Information for
Tuning the Fröhlich exciton-phonon scattering in monolayer MoS_2

B Miller et al.

Supplementary Note 1: Field effect devices with electrolyte top gate

Figure S1(a) shows a scheme of a field effect device we use for Raman and PL spectroscopy in dependence of the free electron density. We use a PDMS stamping technique [1] to transfer monolayer MoS_2 flakes onto silicon substrates with a 300 nm thick SiO_2 layer as dielectric. Contacts to the MoS_2 flake and for the electrolyte top gate are fabricated by standard optical lithography and e-beam evaporation of 5 nm Ti and 30 nm Au. As an electrolyte top gate we use either a solid polymer electrolyte (PE) of poly-(ethylene oxide) and $CsClO_4$ or the ionic liquid (IL) Diethyl-methyl-(2-methoxyethyl)-ammonium-bis-(trifluormethylsulfonyl)-imid (Sigma Aldrich). In both cases, the gating principle relies on the separation of ions in the electrolyte and the formation of an electronic double layer between the ions of the electrolyte and the two-dimensional material. The data shown in Fig. 2 of the manuscript origin from a field effect device with PE top gate [sample A]. The capacity of PE top gates is in the order of μF, two orders of magnitude higher than the capacity of the silicon back gate [2, 3]. For monolayer MoS_2, Chakraborty *et al.* reported a modification of the charge carrier density of $\sim 10^{13}$ cm^{-2} for an applied gate voltage of $V_{\mathrm{TG}} = 1$ V in a PE field effect device [3]. Further, the authors demonstrated that the energy of the A_1' phonon mode is sensitive to the electron density and they correlated the energy shift of the A_1' mode to a change of the electron density. As a determination of the absolute charge carrier density in the MoS_2 flake is beyond the scope of our devices, we use ref. 3 to estimate the change of the charge carrier density from the energy shift of the A_1' mode measured by Raman spectroscopy. Supplementary Figure 1(c) shows non-resonant Raman spectra in the circular co-polarized configuration for two gate voltages $V_{\mathrm{TG}} = -0.5$ V (corresponding to n^0 of sample A with PE gate) and $V_{\mathrm{TG}} = 0$ V (corresponding to n^{++} of sample A with PE gate). Lorentzian fits to the data reveal energies of 405.5 cm^{-1} and 403.3 cm^{-1} for the A_1' mode, respectively. The shift of 2.2 cm^{-1} corresponds to a change of the electron density of $\sim 10^{13}$ cm^{-2} according to ref. [3]. For n^0, we assume an electron density in the order of $\sim 10^{11}$ cm^{-2}, what is supported by the comparison of our PL spectra to the spectra shown in ref. [4]. Overall, the estimation results in a ratio of $\mathrm{n}^{++}/\mathrm{n}^0 \sim 100$. In order to avoid asymmetric electric fields in the MoS_2 flake, we simultaneously use the electrolyte top gate and the silicon back gate with a ratio of $V_{\mathrm{BG}}/V_{\mathrm{TG}} = 80$. We would like to note, however, that an asymmetric field does not affect the conclusions drawn in the manuscript [*cf.* Supplementary Figure 6].

One peculiarity of the PE is its helical crystallization. Therefore, it can cause changes in the degree of circular polarization by turning circular polarized light into linear polarized light and by causing depolarization. For this reason, the measurements on the PE gate were conducted in the configuration shown in Fig S1(b), which makes it possible to monitor the degree of circular polarization during the measurements individually for each device since the change in the degree of circular polarization caused by the PE gate changes from sample to sample.

In particular, it is possible to measure the effect of the PE gate by comparing the reflected laser light from two spots on the bare silicon substrate and on silicon substrate covered with PE. The measurements are shown in the first two rows of Supplementary Figure 1(e). The polarization dependence of the intensities of the Raman signal can be corrected for the effect of the PE gate by deconvolution. The polarization dependence of the fitted amplitudes of the A_1' and the E' modes is shown in Supplementary Figure 1(e) before and after the deconvolution of the effect of the PE gate. The deconvoluted data corresponds to the data shown in Fig. 2(f)-(g) of the manuscript.

The data shown in Fig. 1 of the manuscript origins from a field effect device with IL top gate [sample B]. ILs are widely used to modulate the carrier density in two-dimensional materials and they provide capacities comparable to those of PE gates [5, 6]. Supplementary Figure 1(d) shows non-resonant Raman spectra from a field effect device with IL top gate [sample B] in the circular co-polarized configuration for two gate voltages $V_{\mathrm{TG}} = -2\,\mathrm{V}$ and $V_{\mathrm{TG}} = 1\,\mathrm{V}$ (corresponding to n^0 and n^{++} of sample B with IL gate, *cf.* Fig. 1 of the manuscript). Lorentzian fits to the data reveal energies of $403.8\,\mathrm{cm}^{-1}$ and $400.2\,\mathrm{cm}^{-1}$ for the A_1' mode, respectively. The shift of $3.6\,\mathrm{cm}^{-1}$ verifies an equally high capacity of the IL top gate.

Supplementary Note 2: Matrix element of the Fröhlich exciton-phonon interaction

The Fröhlich exciton-phonon interaction for an exciton is derived by combining the electron-phonon Fröhlich interaction of an electron and a hole [7, 8]:

$$H_{\mathrm{FI}} = \frac{C_{\mathrm{F}}}{q}\left(\frac{1}{[1+(p_{\mathrm{h}}a_0q/2)^2]^2} - \frac{1}{[1+(p_{\mathrm{e}}a_0q/2)^2]^2}\right) \tag{1}$$

$$C_{\mathrm{F}} = e\left[\frac{2\pi\hbar\omega_{\mathrm{LO}}}{NV}\left(\varepsilon_\infty^{-1} - \varepsilon_0^{-1}\right)\right]^{1/2}, \quad p_{\mathrm{e}} = \frac{m_{\mathrm{e}}}{m_{\mathrm{e}}+m_{\mathrm{h}}} \quad \text{and} \quad p_{\mathrm{h}}\frac{m_{\mathrm{h}}}{m_{\mathrm{e}}+m_{\mathrm{h}}} \tag{2}$$

For the electron and hole masses we use $m_{\mathrm{e}} = 0.46$ and $m_{\mathrm{h}} = 0.54$ [9]. For the exciton radius a_0 we assume $a_0 = 1\,\mathrm{nm}$ [10]. C_{F} contains the frequency of the LO phonon ω_{LO}, the volume V, the number of unit cells per unit volume N and the high and low frequency dielectric constants ε_∞ and ε_0. For the plot shown in Fig. 3(a) of the manuscript we assume C_{F} to be a constant and set it arbitrarily to 10^{-4}.

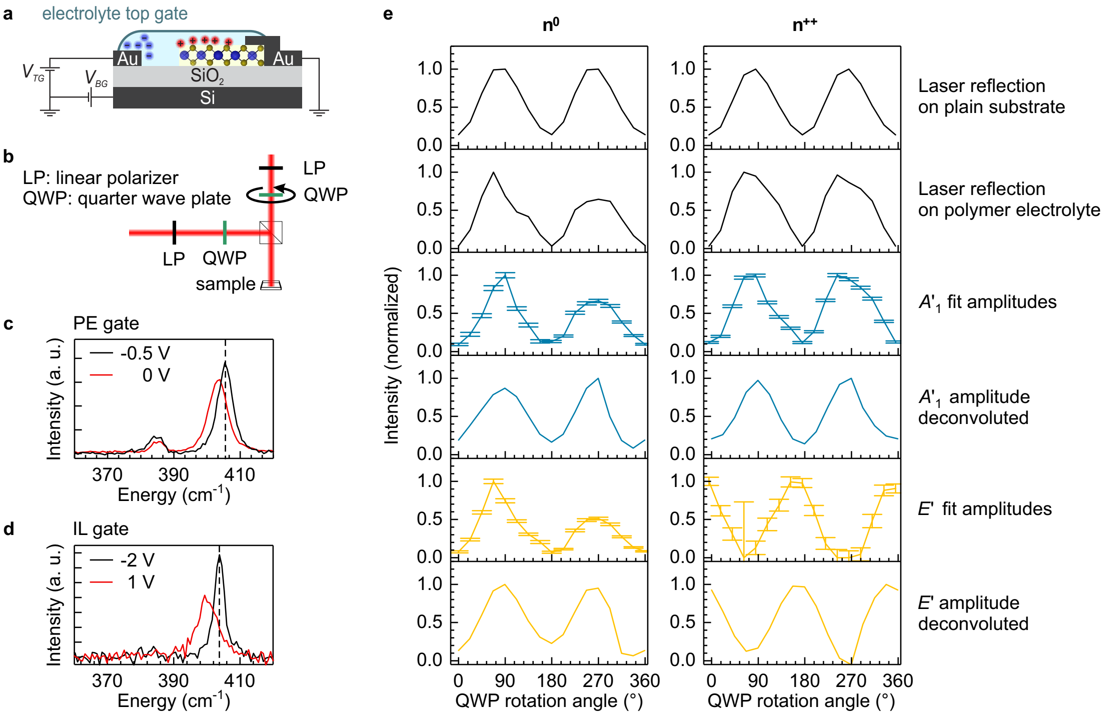

Supplementary Figure 1 — Characterization of field effect devices. (a) Scheme of a field effect device used for optical measurements in dependence of the free electron density. (b) Scheme for polarization resolved measurements on devices with PE gate. (c) Non-resonant Raman spectra ($E_\mathrm{i} = 2.54$ eV) in circular co-polarized configuration for two top gate voltages of a device with PE gate [sample A]. (d) Non-resonant Raman spectra ($E_\mathrm{i} = 2.54$ eV) in circular co-polarized configuration for two top gate voltages of a device with IL gate [sample B]. (e) Deconvolution of the polarizing effect of the PE top gate [sample A]. The left panel shows data for low electron density n^0, the right panel shows data for high electron density n^{++}. The error bars in the plots of the fitted amplitudes represent the fit errors.

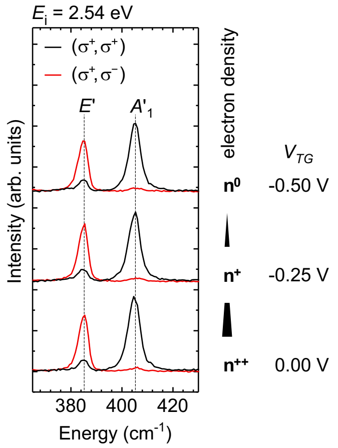

Supplementary Figure 2 — Gate dependence of non-resonant Raman spectra. Non-resonant Raman spectra ($E_\mathrm{i} = 2.54\,\mathrm{eV}$) in circular co-polarized (black) and cross-polarized (red) configurations for electron densities n^0, n^+ and n^{++}. Data taken on sample A with PE top gate.

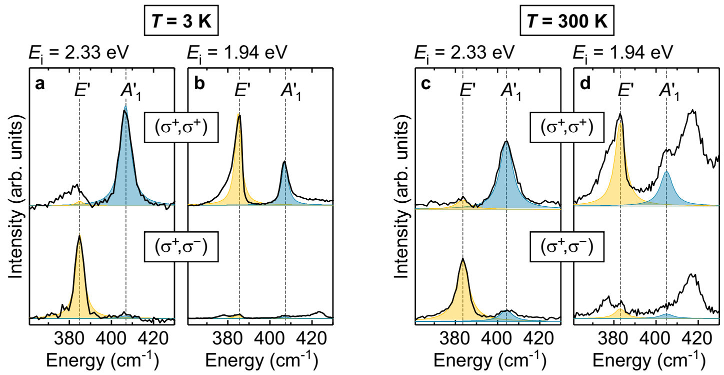

Supplementary Figure 3 — Temperature dependence of Raman spectra of CVD grown $\mathbf{MoS_2}$. Circular co-polarized (σ^+,σ^+) and cross-polarized (σ^+,σ^-) Raman spectra of a CVD grown monolayer MoS_2 flake on a SiO_2/Si substrate. Black lines represent measured spectra; filled curves are Lorentzian fits to the data. (a) Low temperature and non-resonant excitation, (b) low temperature and resonant excitation, (c) room temperature and non-resonant excitation and (d) room temperature and resonant excitation. The polarization dependence is qualitatively the same for both temperatures and it matches the data from exfoliated flakes for the case of low electron densities shown in the main part of the manuscript. We note that exfoliated MoS_2 monolayers can intrinsically be in the low or high electron density regime. This large variation in the intrinsic charge carrier density from sample to sample might explain conflicting reports in literature for pristine MoS_2 monolayers demonstrating the E' phonon being cross-polarized [11] or co-polarized [12] under resonant excitation.

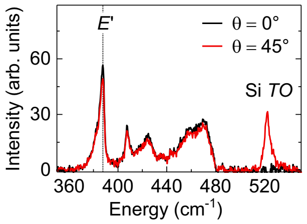

Supplementary Figure 4 — Linear polarized Raman spectra. Resonant Raman spectra ($E_\mathrm{i} = 1.96\,\mathrm{eV}$) for two different angles θ between the crystal axes of the sample and the polarization direction of the linear polarized incident and scattered light. Incident and scattered light are linear parallel polarized. Fitted amplitudes are shown in Fig. 3(c) of the manuscript.

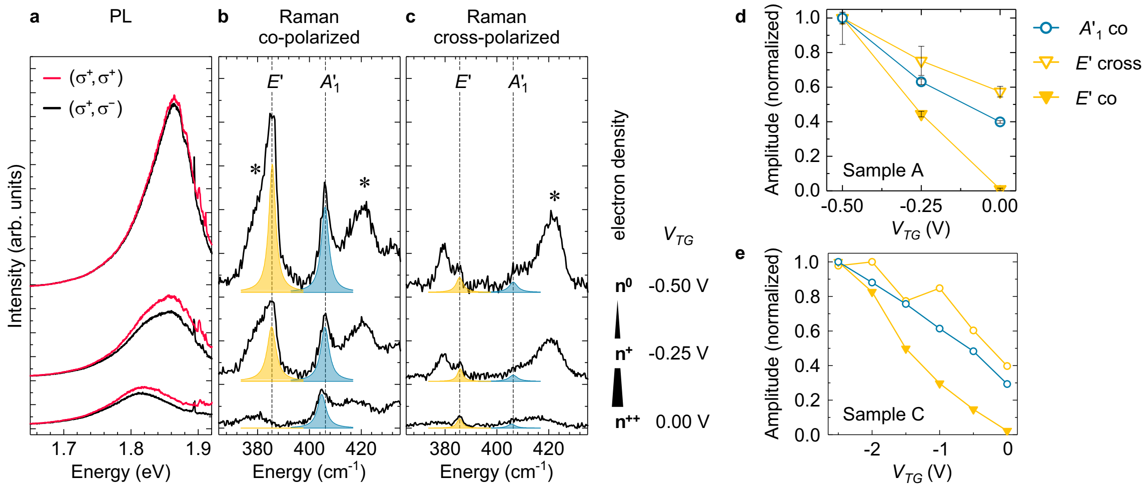

Supplementary Figure 5 — Gate dependence of PL and resonant Raman spectra. PL and resonant Raman spectra taken at $T = 300\,\mathrm{K}$ with a laser energy of ($E_\mathrm{i} = 1.96\,\mathrm{eV}$) for three different electron densities n^0, n^+ and n^{++}. (a) PL spectra in the circular co- (red) and cross-polarized configuration. (b) Circular co-polarized and (c) cross-polarized resonant Raman spectra. Filled curves show fitted Lorentzian peaks for the A_1' and E' modes. (d, e) Fitted amplitudes of the Raman and PL intensities in dependence of the top gate voltage. DP contributions ($A'_{1\mathrm{CO}}$ and E'_{CROSS}) are plotted as open circles, the FI contribution (E'_{CO}) as filled triangles. The error bars represent the fit errors. Data is shown for two samples with PE top gate. Data shown in panels (a-d) as well as data in Supplementary Figure 1(c,e), Supplementary Figure 2, Supplementary Figure 6 and Fig. 2 of the manuscript is taken on sample A.

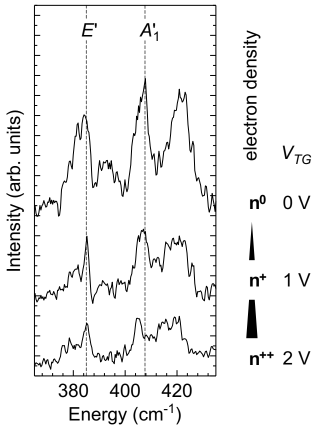

Supplementary Figure 6 — Gate dependence of Raman spectra for positive gate voltages. Resonant Raman spectra taken at $T = 300\,\mathrm{K}$ with a laser energy of ($E_\mathrm{i} = 1.96\,\mathrm{eV}$) for three different positive gate voltages on a sample with IL top gate. For $V_\mathrm{TG} = 0$ V we observe strong scattering from the LO mode E', while the scattering rate decreases for increasing positive gate voltages. In contrast, for sample A we observe that the LO scattering is suppressed for $V_\mathrm{TG} = 0$ V and is strongly enhanced for negative voltages [*cf.* Supplementary Figure 5]. The difference between the two samples can be explained by different intrinsic doping levels due to the exfoliation process. Due to the contrasting behavior of the two samples when increasing the absolute of the gate voltage, we exclude symmetry breaking by the electric field of the gate to be responsible for the observation of strong co-polarized LO scattering. This conclusion is consistent to the observation of strong co-polarized LO scattering in samples without any top gate as shown in Supplementary Figure 3.

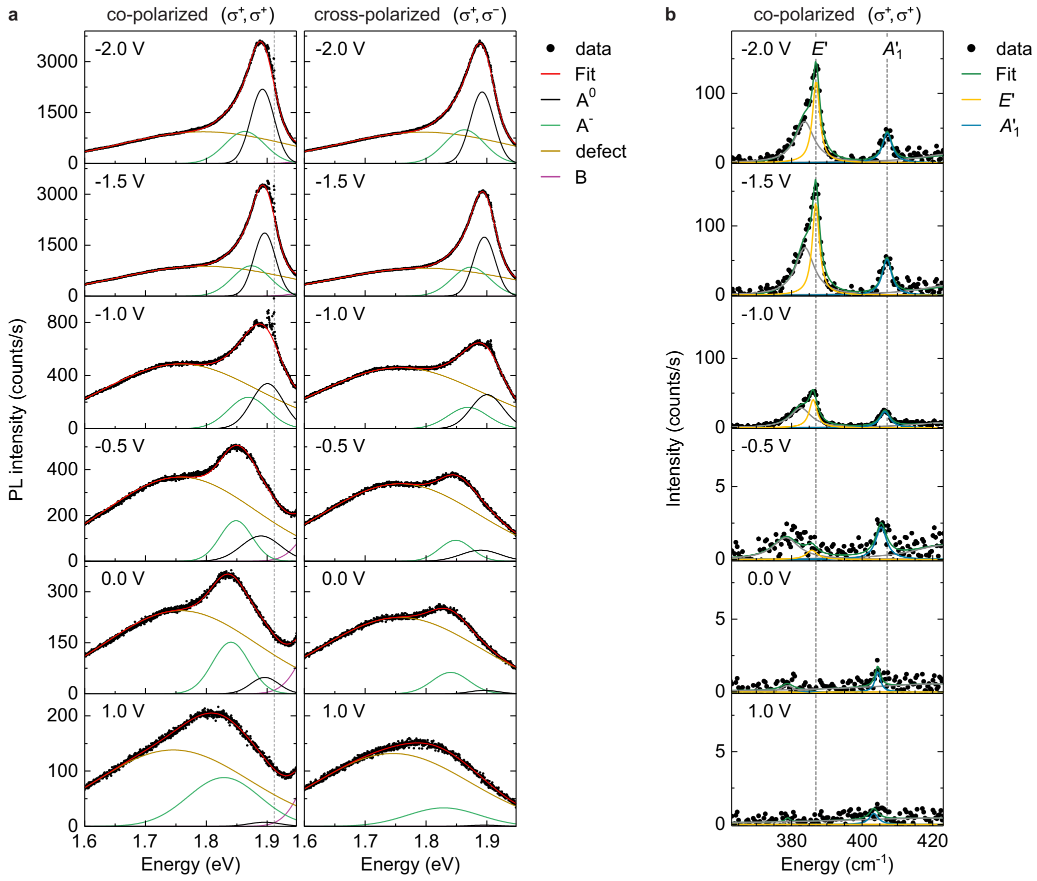

Supplementary Figure 7 — Line-shape analysis of PL and Raman spectra. (a) Circular co- and cross-polarized PL spectra (left and right panel of (a), respectively) in dependence of the applied top gate voltage [data also shown in Fig. 1 of the manuscript]. The plots show the original data (black scatter) together with a multi-peak fit consisting of four Gaussian peaks that represent the PL of the neutral and charged A exciton (A^0 and A^-), the B exciton and a defect peak. For increasing gate voltage from -2 V to 1 V we observe a bleaching of both the neutral and the charged exciton emission. The A^0 peak broadens from ≈ 45 meV to ≈ 70 meV and its energy slightly blue shifts by $\approx 4\,\text{meV} \pm 10\,\text{meV}$. The A^- peak redshifts by 35 meV, corresponding to half of its full width at half maximum of ≈ 70 meV. The trends are consistent to existing literature for MoS_2 [4] and other TMDs [13]. The dashed line indicates the energy of light scattered by the E' phonon and shows that the resonance condition for the light scattering is satisfied for all gate voltages. (b) Circular co-polarized Raman spectra corresponding to the spectra PL spectra shown in (a). Measured data is shown as black scatters. Solid lines are Lorentzian peaks fitted to the data. Grey lines are peaks of resonant Raman modes that are discussed in literature. In ciruclar co-polarized configuration the A_1' mode is a contribution due to the deformation potential, whereas we ascribe the E' mode contribution [yellow line] to the Fröhlich interaction. For increasing gate voltages we observe decreasing intensities for both contributions, however the intensity of the E' mode decreases faster than that of the A_1' mode [intensity ratio plotted in Supplementary Figure 9(c)].

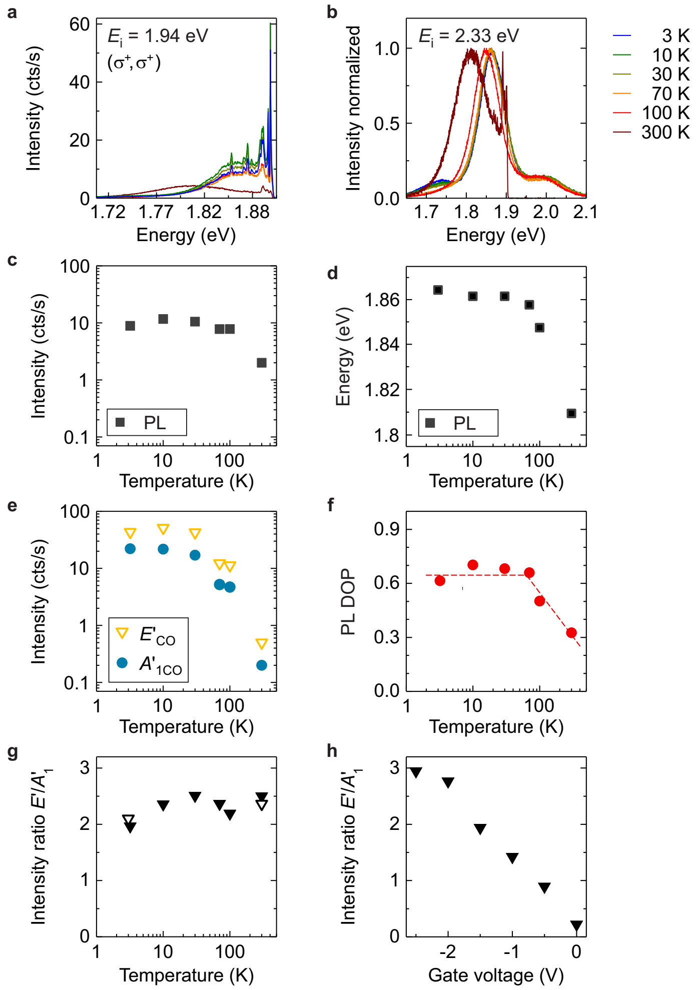

Supplementary Figure 8 — Temperature dependence of PL and resonant Raman spectra. Temperature dependent spectra of CVD grown monolayer MoS_2 [*cf.* Supplementary Figure 3]. (a) Circular co-polarized spectra for resonant excitation at $E_\mathrm{i} = 1.94\,\mathrm{eV}$ (b) Normalized spectra for excitation at $E_\mathrm{i} = 2.33\,\mathrm{eV}$. The spectrum for 300 K is recorded with $E_\mathrm{i} = 1.94\,\mathrm{eV}$. (c) Logarithmic plot of the PL intensity in dependence of the temperature extracted from the resonant spectra shown in (a). (d) Center energy of the PL peak in dependence of the temperature extracted from the spectra shown in (b). (e) Temperature dependence of the intensity of the co-polarized Raman contributions E'_CO and A'_1CO extracted from the resonant spectra in (a). The intensity of both modes decreases with increasing temperature. This effect is consistent to the bleaching and the shift of the excitonic resonance [shown in (c) and (d), respectively]. (f) Temperature dependence of the degree of polarization (DOP) of the PL spectra shown in (a). The DOP decreases monotonously with increasing temperature. (g) Ratio of the intensities of E'_CO and A'_1CO plotted in (e). The intensity ratio is constant over the whole temperature range, demonstrating that the temperature dependent bleaching and shift of the resonance equally affects the scattering probabilities of the DP contribution A'_1CO and the Fröhlich contribution E'_CO. (h) Gate voltage dependence of the ratio of the intensities of E'_CO and A'_1CO of sample C with PE top gate [*cf.* Supplementary Figure 5(e)]. The data shows that the intensity of E'_CO decreases much faster than the intensity of A'_1CO with increasing electron density. From the temperature dependence shown in (g) we can exclude that the gate dependence of the resonance condition is responsible for the faster suppression of the E' mode. Therefore, we conclude that this effect is caused by screening of the Fröhlich scattering with increasing electron density.

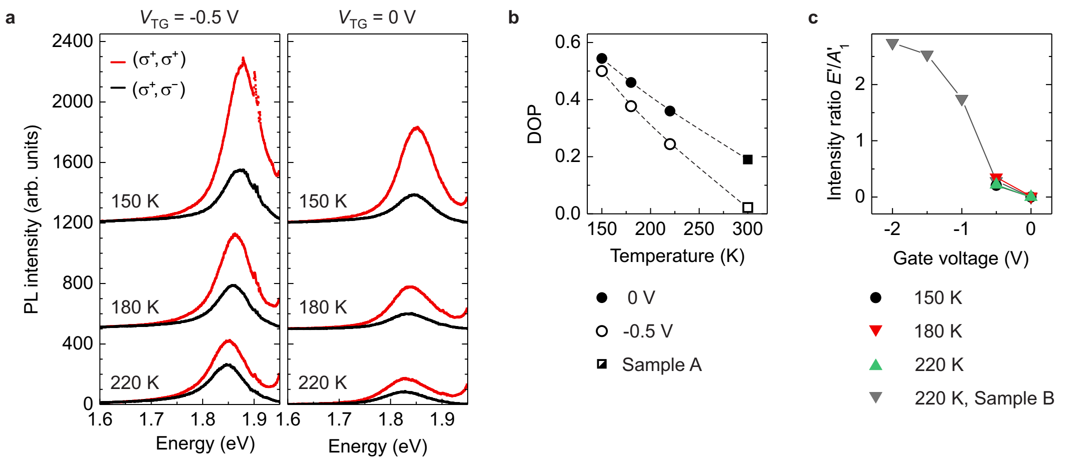

Supplementary Figure 9 — Temperature dependence of the degree of polarization. (a) Temperature series of co- and cross-polarized PL spectra of a monolayer MoS_2 device with IL top gate for two different electron densities. (b) Degree of polarization (DOP) of the spectra shown in (a) evaluated at the respective maxima of the PL peak (circles). Squares represent the DOP of the PL spectra shown in Supplementary Figure 5 [sample A]. Dashed lines are guides to the eyes. (c) Intensity ratio of the E' and the A_1' Raman modes of circular co-polarized spectra. Grey triangles represent data taken on sample B [sample of Fig. 1 of the manuscript, spectra shown in Supplementary Figure 7(b)].

Supplementary References

[1] Castellanos-Gomez, A. *et al.* Deterministic transfer of two-dimensional materials by all-dry viscoelastic stamping. *2D Materials* **1**, 011002 (2014).

[2] Das, A. *et al.* Monitoring dopants by Raman scattering in an electrochemically top-gated graphene transistor. *Nature Nanotechnology* **3**, 210 (2008).

[3] Chakraborty, B. *et al.* Symmetry-dependent phonon renormalization in monolayer MoS_2 transistor. *Physical Review B* **85**, 161403 (2012).

[4] Mak, K. F. *et al.* Tightly bound trions in monolayer MoS_2. *Nature Materials* **12**, 207 (2013).

[5] Ye, J. T. *et al.* Superconducting Dome in a Gate-Tuned Band Insulator. *Science* **338**, 1193 (2012).

[6] Costanzo, D., Jo, S., Berger, H. & Morpurgo, A. F. Gate-induced superconductivity in atomically thin MoS_2 crystals. *Nature Nanotechnology* **11**, 339 (2016).

[7] Yu, P. Y. Excitons. In Cho, K. (ed.) *Excitons*, vol. 14 of *Topics in Current Physics*, chap. 5, 211 (Springer Berlin Heidelberg, Berlin, Heidelberg, 1979).

[8] Yu, P. Y. & Cardona, M. *Fundamentals of Semiconductors*. Graduate Texts in Physics (Springer Berlin Heidelberg, Berlin, Heidelberg, 2010).

[9] Kormányos, A. *et al.* k.p theory for two-dimensional transition metal dichalcogenide semiconductors. *2D Materials* **2**, 49501 (2015).

[10] Berkelbach, T. C., Hybertsen, M. S. & Reichman, D. R. Theory of neutral and charged excitons in monolayer transition metal dichalcogenides. *Physical Review B* **88**, 045318 (2013).

[11] Chen, S. Y., Zheng, C., Fuhrer, M. S. & Yan, J. Helicity-Resolved Raman Scattering of MoS_2, $MoSe_2$, WS_2, and WSe_2 Atomic Layers. *Nano Letters* **15**, 2526 (2015).

[12] Drapcho, S. G. *et al.* Apparent breakdown of Raman selection rule at valley exciton resonances in monolayer MoS_2. *Physical Review B* **95**, 165417 (2017).

[13] Chernikov, A. *et al.* Electrical Tuning of Exciton Binding Energies in Monolayer WS_2. *Physical Review Letters* **115**, 126802 (2015).

[14] Zeng, H., Dai, J., Yao, W., Xiao, D. & Cui, X. Valley polarization in MoS_2 monolayers by optical pumping. *Nature Nanotechnology* **7**, 490–493 (2012).

[15] Luo, X., Zhao, Y., Zhang, J., Xiong, Q. & Quek, S. Y. Anomalous frequency trends in MoS_2 thin films attributed to surface effects. *Physical Review B* **88**, 075320 (2013).

[16] Livneh, T. & Spanier, J. E. A comprehensive multiphonon spectral analysis in MoS_2. *2D Materials* **2**, 035003 (2015).
